# Quantification of sigmoid perfusion with near-infrared fluorescence and indocyanine green during open abdominal aortic aneurysm reconstruction

**DOI:** 10.1016/j.jvscit.2026.102336

**Published:** 2026-05-26

**Authors:** Roderick C. Peul, Mo W. Kruiswijk, Stefan Koning, Louelle E. van der Aa, Indy Planting, Jeroen L. Umbgrove, Floris P. Tange, Pim van den Hoven, Jeroen J.W.M. Brouwers, Abbey Schepers, Jaap F. Hamming, Alexander L. Vahrmeijer, Jan van Schaik, Joost R. van der Vorst

**Affiliations:** Department of Surgery, Leiden University Medical Center, Leiden, the Netherlands

**Keywords:** Colon ischemia, Open abdominal aortic aneurysm repair, Near-infrared fluorescence imaging, Indocyanine green, Perfusion, Quantification

## Abstract

**Objective:**

This study evaluated the feasibility of quantitative near-infrared fluorescence imaging with indocyanine green (NIRF-ICG) to assess changes in colon perfusion induced by abdominal aortic aneurysm reconstruction. Improved perfusion assessment may support clinical decision-making in patients at risk for developing colon ischemia.

**Methods:**

NIRF-ICG recordings of the sigmoid colon from 25 patients treated at a single center were retrospectively analyzed. Time-intensity curves were extracted from the sigmoid colon and small intestine and classified as demonstrating adequate, questionable, or poor perfusion. Four curve-derived perfusion parameters (time to maximum intensity, maximum inflow rate of the normalized curve, and remaining fluorescence intensity 30 seconds and 60 seconds after maximum intensity) were compared to objectively evaluate changes in fluorescence inflow and outflow before and after aortic reconstruction.

**Results:**

Quantitative analysis was feasible in 23 patients undergoing elective procedures and in 1 patient undergoing acute surgery. Although all patients demonstrated adequate perfusion on clinical assessment, quantitative classification identified perfusion deterioration in 13 patients. All four derived perfusion parameters showed a significant decrease in sigmoid perfusion after aortic reconstruction (time to maximum intensity, *P* < .001; maximum inflow rate of the normalized curve, *P* = .019; remaining fluorescence intensity 30 seconds after maximum intensity, *P* = .029; remaining fluorescence intensity 60 seconds after maximum intensity, *P* = .021), whereas small intestinal perfusion remained unchanged (all *P* > .05). In two cases, severely impaired perfusion detected by NIRF-ICG imaging recovered postoperatively, as confirmed by a NIRF-ICG measurement during planned second-look surgery. Two patients classified as having poor perfusion required acute relaparotomy due to anastomotic bleeding. There were no cases of colon ischemia, and mortality was zero.

**Conclusions:**

Quantitative NIRF enables objective intraoperative assessment of sigmoid perfusion during open abdominal aortic aneurysm repair. The observed recovery from transient perfusion deficits supports heightened awareness rather than immediate bowel resection in cases suspected of malperfusion. Validation of perfusion patterns predictive of ischemia is required to establish risk stratification criteria and optimize clinical decision-making.

**Article Highlights**

• **Type of Research:** Single-center retrospective cohort study
• **Key Findings:** Quantitative near-infrared fluorescence imaging with indocyanine green enables objective sigmoid colon perfusion assessment during open abdominal aortic aneurysm repair. Despite significant perfusion deterioration after reconstruction (4/4 perfusion parameters with a significant decline), the absence of colon ischemia in 24 patients supports heightened awareness rather than immediate bowel resection in cases of suspected malperfusion.
• **Take Home Message:** Quantitative near-infrared fluorescence imaging with indocyanine green provides the surgical team with an objective, real-time assessment of sigmoid colon perfusion during open abdominal aortic aneurysm repair.

Open surgical reconstruction is a crucial intervention in the management of patients with an abdominal aortic aneurysm (AAA), but is associated with significant perioperative risks.^1^^,^^2^ Colon ischemia (CI) occurs in ≤4.2% of elective surgeries, with a higher incidence reported for acute cases.^3^, ^4^, ^5^ Mortality among patients who develop CI exceeds 50%.^6^^,^^7^ Ischemia most frequently occurs at Sudeck's point, a watershed area between the inferior mesenteric artery (IMA) and the pelvic circulation.^8^ This makes the sigmoid colon particularly vulnerable to perfusion disturbances following vasopressor use and IMA ligation during aortic reconstruction.^9^^,^^10^

Reimplantation of the IMA has not demonstrated superiority over conventional surgery in preventing CI.^11^ Moreover, it is associated with prolonged surgery duration, an increased risk of reintervention, and a higher incidence of wound complications.^12^ Consequently, no effective methods currently exist to reliably reduce the risk of CI, making early detection critically important. The most commonly used intraoperative method to assess colon perfusion involves visual inspection of intestinal color and pulsation of small mesenteric vessels. However, this approach is highly dependent on the subjective interpretation of the surgeon. Postoperative monitoring relies primarily on clinical signs, and, in cases of suspected CI, flexible sigmoidoscopy is recommended.^13^^,^^14^ Although this modality allows evaluation of the mucosal surface, it does not assess transmural ischemia. Furthermore, at the moment of evaluation, ischemia has been present for a prolonged period, increasing mortality risk. Collectively, these limitations highlight the need for an objective intraoperative tool capable of identifying patients at increased risk for CI.

Near-infrared fluorescence imaging with indocyanine green (NIRF-ICG) has proven to be a safe and effective intraoperative modality for detecting malperfusion during colon resection surgery.^15^^,^^16^ However, large-scale studies have relied primarily on qualitative interpretations of fluorescence signal differences. Qualitative assessment presents challenges when applying this technique to assess colon perfusion during AAA repair, as comparisons of NIRF-ICG measurements have to be made between measurements taken before and after aortic reconstruction, often separated by several hours. Quantitative analysis of fluorescence signal intensity over time might offer a solution to this limitation.^17^^,^^18^ By generating objective time-intensity curves and extracting relevant perfusion parameters, it becomes possible to perform more reliable comparisons of colon perfusion before and after aortic reconstruction.^19^

This study evaluated the potential of quantitative NIRF-ICG imaging to characterize changes in perfusion patterns before and after aortic reconstruction. Such assessments may provide valuable insights into identifying patients at risk for CI and support intraoperative and postoperative decision-making.

## Methods

### Design

This is a retrospective feasibility study on quantitative NIRF-ICG imaging for perfusion assessment of the sigmoid colon during open AAA reconstruction. Quantification was performed postoperatively on NIRF-ICG recordings acquired as standard of care during surgery. Approval was granted by the non-WMO Committee and the Research and Science Committee of the Department of Surgery, Leiden University Medical Center (2024-015).

### Study population

The study population consisted of 25 patients undergoing open AAA reconstruction with a NIRF-ICG measurement in the Leiden University Medical Center between September 2023 and January 2026.

### Data acquisition

Intraoperative perfusion of the sigmoid colon was assessed using qualitative NIRF-ICG imaging before and after aortic reconstruction with a dedicated NIRF camera (Quest Spectrum Platform, Quest Medical Imaging). Measurement after reconstruction was performed before abdominal closure to allow for the longest possible reperfusion period. All measurements meeting quantification criteria were included in this study. These criteria included a fixed, standardized camera position and distance toward the tissue, adequate visualization of the sigmoid colon within the field of view, and a fixed ICG dosage administered via a peripheral venous line. Furthermore, perioperative medical records were collected to analyze patient characteristics, vital signs, blood loss, duration of hospital stay, and postoperative complications.

### Quantification

A quantitative analysis of the NIRF-ICG measurements was performed using the Quest Research Framework (Quest Medical Imaging). With this software platform, time-intensity curves were generated for regions of interest encompassing the entire sigmoid colon, with additional proximal, middle, and distal regions of interest. When possible, a control region of interest was placed on the small intestine to account for systemic influences on perfusion (Supplementary Fig, *A*, online only). Motion-related signal fluctuations were corrected using a discriminative correlation filter with channel and spatial reliability.^20^

For each region of interest, normalized time-intensity curves were plotted. Normalization to maximum intensity was used to account for measurement-related variability and improve comparability across patients.^21^^,^^22^ Curves were categorized as adequate, questionable, or poor perfusion according to the colon perfusion classification described by Faber et al.^17^

Four different perfusion parameters were derived from the curves (Supplementary Fig, *B*, online only). Inflow parameters consisted of time to maximum intensity (Tmax [seconds]) and maximum inflow rate of the normalized curve (MaxSlope [%/s]). Outflow parameters included remaining fluorescence intensity 30 seconds and 60 seconds after maximum intensity (AUC30 and AUC60 [%]).

### Analysis

Time-intensity curves were visualized using GraphPad Prism version 10.0.0 (GraphPad). Statistical analyses were performed with IBM SPSS Statistics 29 (IBM). Descriptive statistics were used to summarize patient characteristics and perfusion parameter values before and after aortic reconstruction. To evaluate perfusion changes between measurements before and after aortic reconstruction, Wilcoxon signed-rank tests were performed for individual parameters changes and independent-samples *t*-tests and Mann-Whitney *U* tests for comparisons between groups.

## Results

### Patient characteristics

NIRF-ICG recordings of 25 patients were present, with one case excluded due to ICG administration via a central venous line instead of a peripheral line, resulting in 24 NIRF-ICG recordings eligible for quantitative analysis. In seven patients, the surgery included graft explantation of a previous AAA reconstruction (five endovascular aneurysm repairs [EVARs], one fenestrated EVAR [FEVAR], and one open aortic graft). One patient underwent reconstruction of a ruptured AAA, meaning that NIRF-ICG imaging was only performed after aortic reconstruction. The mean patient age was 70.6 ± 7.4 years, with 25% female patients. In six patients, the IMA was patent. A further six patients suffered from iliac artery stenosis. One patient had a short-segment superior mesenteric artery (SMA) occlusion, and another had a 50% SMA stenosis. During surgery, infrarenal cross-clamping was performed in 18 patients, and 4 required suprarenal clamping. One patient underwent diagonal cross-clamping (left suprarenal, right infrarenal). In another, initial suprarenal clamping was converted to infrarenal following EVAR explantation. No patient underwent IMA reimplantation. In 12 patients, the small intestine could be analyzed alongside the sigmoid colon. Patient characteristics are summarized in Table I.

**Table I:** Patient characteristics (n = 24)

characteristics	Value
Age, years	70.6 ± 7.4
Female sex	6 (25.0)
BMI, kg/m^2^	26.0 ± 3.9
Smoking	
No	5 (20.8)
Former	12 (50.0)
Active	5 (20.8)
Unknown	2 (8.3)
Comorbidities	
Hypertension	15 (62.5)
Coronary artery disease	3 (12.5)
Peripheral artery disease	5 (20.8)
Diabetes	2 (8.3)
Vascular characteristics	
Aneurysm diameter, mm	61 (54-98)
IMA patency	6
SMA stenosis	2
Iliac artery stenosis	6
Type of surgery	
Open AAA reconstruction	16 (66.7)
Graft explantation after EVAR	4 (16.7)
Graft explantation after open reconstruction	1 (4.2)
Nevelsteen procedure	1 (4.2)
Graft explantation after FEVAR	1 (4.2)
Ruptured AAA reconstruction	1 (4.2)
ASA score	
2	9 (37.5)
3	11 (45.8)
4	3 (12.5)
5	1 (4.2)
Previous abdominal surgery	9 (37.5)
Surgery duration, minutes	213 (129-694)
Length of stay, days	7.5 (4-42)

*AAA*, Abdominal aortic aneurysm; *ASA*, American Society of Anesthesiologists; *BMI*, body mass index; *EVAR*, endovascular aneurysm repair; *FEVAR*, fenestrated endovascular aneurysm repair; *IMA*, inferior mesenteric artery; *SMA*, superior mesenteric artery.

Values are mean ± standard deviation, number (%), or median (range).

### Intraoperative perfusion assessment

**Fig 1:**
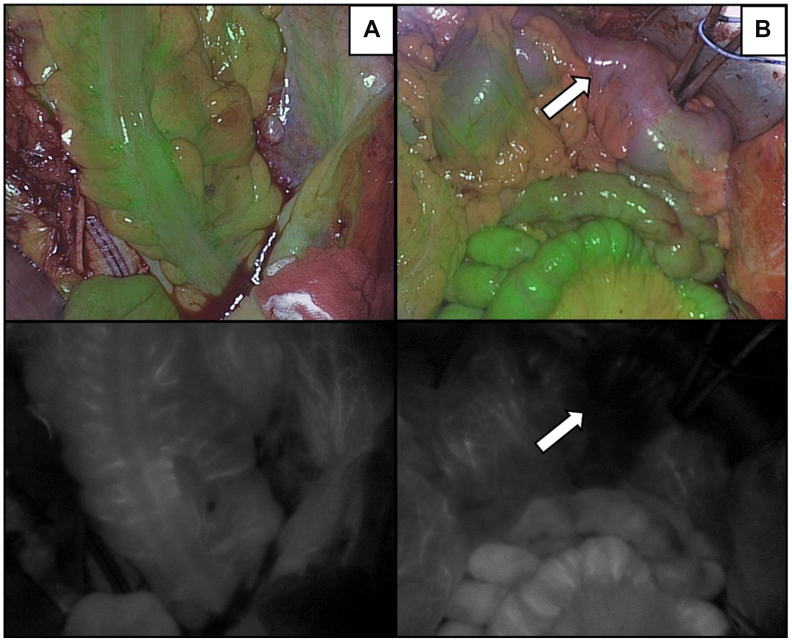
Qualitative near-infrared fluorescence imaging with indocyanine green (NIRF-ICG) imaging of the sigmoid colon after aortic reconstruction. **(A)** Patient demonstrating adequate perfusion of the entire visible sigmoid colon. **(B)** Patient with absence of fluorescence signal in the area marked by the *white arrows*, indicating poor perfusion.

Before aortic reconstruction, both clinical perfusion assessment and qualitative NIRF-ICG imaging of sigmoid perfusion were considered adequate in all cases. After reconstruction, clinical assessment remained adequate in all patients. However, in six patients, qualitative interpretation of the NIRF-ICG signal by the surgical team indicated deterioration of perfusion. Of these six patients, four were reported to have questionable perfusion deterioration, characterized by reduced fluorescence intensity and a slower inflow rate. Two patients were qualitatively classified as having poor perfusion compared with the baseline measurement, with minimal to absent fluorescence signal observed in portions of the sigmoid colon (adequate perfusion [Fig 1, *A*] and poor perfusion [Fig 1, *B*]). All segments of the small intestine demonstrated adequate perfusion according to both clinical evaluation and qualitative NIRF-ICG assessment. The mean arterial pressure was significantly higher after aortic reconstruction.

### Quantitative NIRF-ICG analysis

**Fig 2:**
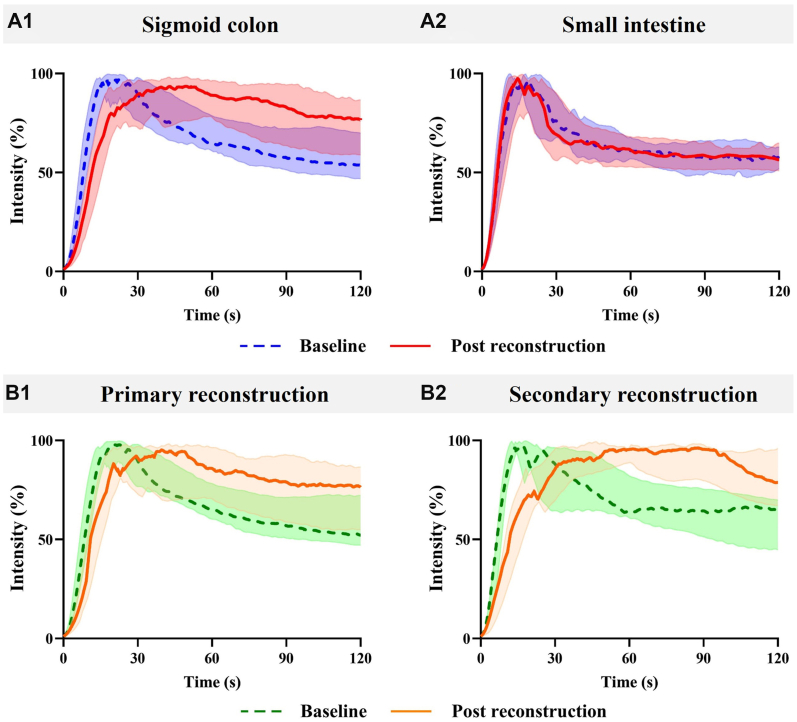
Median time-intensity curves before and after aortic reconstruction. **(A)** Time-intensity curves of the sigmoid colon **(A1)** and small intestine **(A2)**. **(B)** Time-intensity curves of the sigmoid colon during primary reconstruction **(B1)** and secondary reconstruction **(B2)**.

Of the 24 recordings eligible for quantitative analysis, time-intensity curves were successfully extracted from the sigmoid colon and classified. Before aortic reconstruction, all but one case demonstrated an adequate perfusion pattern, with the remaining case classified as questionable. After aortic reconstruction, 11 cases were categorized as exhibiting adequate perfusion, 9 as questionable perfusion, and 4 as poor perfusion. A significant difference in perfusion curves and median parameter values was seen before and after aortic reconstruction (Tmax, *P* < .001; MaxSlope, *P* = .019; AUC30, *P* = .029; AUC60, *P* = .021) as visualized in Table II and Fig 2, *A*.

**Table II:** Near-infrared fluorescence imaging with indocyanine green (*NIRF-ICG*) measurement characteristics

	Baseline	Post reconstruction	Δ	*P* value
Diastolic tension, mm Hg	56.5 ± 9.7	59.9 ± 7.3	+6.0%	.077
Mean arterial pressure, mm Hg	74.4 ± 9.8	80.5 ± 9.6	+8.2%	.020^a^
Blood loss, mL	–	1375 (200-7000)	–	–
Clinical perfusion assessment			–	–
Adequate	23	24		
Questionable	0	0		
Poor	0	0		
Qualitative NIRF-ICG perfusion assessment			–	–
Adequate	23	17		
Questionable	0	5		
Poor	0	2		
Curve classification^b^			–	–
Adequate	22	11		
Questionable	1	9		
Poor	0	4		
Perfusion parameters sigmoid colon, median (quartiles)				
Tmax, seconds	16.4 (13.8-21.9)	36.4 (19.6-58.2)	+122.0%	<.001^a^
MaxSlope, %/s	9.9 (8.2-11.4)	7.6 (6.0-8.8)	−23.2%	.019^a^
AUC30, %	83.0 (79.5-90.9)	94.6 (89.1-96.5)	+14.0%	.029^a^
AUC60, %	73.3 (68.8-83.4)	88.5 (80.6-92.9)	+20.7%	.021^a^
Perfusion parameters small intestine, median (quartiles)				
Tmax, seconds	13.4 (9.8-16.9)	13.7 (10.1-16.2)	+2.2%	.374
MaxSlope, %/s	12.7 (11.1-15.8)	13.6 (10.5-14.6)	+7.1%	.477
AUC30, %	76.1 (69.5-81.8)	70.5 (66.1-81.0)	−7.4%	.248
AUC60, %	68.1 (60.9-74.8)	65.2 (58.7-74.0)	−4.3%	.657

*AAA*, Abdominal aortic aneurysm; *ASA*, American Society of Anesthesiologists; *AUC30 and AUC60*, remaining fluorescence intensity 30 seconds and 60 seconds after maximum intensity; *BMI*, body mass index; *EVAR*, endovascular aneurysm repair; *FEVAR*, fenestrated endovascular aneurysm repair; *IMA*, inferior mesenteric artery; *MaxSlope*, maximum inflow rate of the normalized curve; *SMA*, superior mesenteric artery; *Tmax*, time to maximum intensity.

Values are mean ± standard deviation, number (%), or median (range) unless otherwise noted.

a *P* values represents statistical significance (*P* < .05) based on the Wilcoxon signed-rank test.

b Classification according to Faber et al.^17^

The 12 cases in which the small intestine could be analyzed before and after aortic reconstruction demonstrated that perfusion parameters of the small intestine remained unaffected by aortic reconstruction (Table II and Fig 2, *A*) (Tmax, *P* = .374; MaxSlope, *P* = .477; AUC30, *P* = .248; AUC60, *P* = .657). No differences in relative perfusion changes were observed with respect to iliac artery stenosis, SMA stenosis, IMA patency, the level of aortic cross-clamping, or the unclamping-to-measurement duration.

### Quantitative analysis of patients undergoing graft explantation

An analysis of the seven patients undergoing graft explantation (secondary reconstruction) showed a comparable deterioration in perfusion after removal of the previously implanted graft (Fig 2, *B*). Patients undergoing secondary reconstruction had significantly longer operative times (*P* = .044) and greater intraoperative blood loss (*P* = .012).

### Clinical outcomes

In this cohort, no patient developed CI. Sixteen patients experienced postoperative complications within 30 days after surgery. Acute laparotomy was required in two mycotic aneurysm cases. This was due to anastomotic bleeding of the graft in one patient and infected ascites in the other. The patient treated for a ruptured AAA developed acute renal insufficiency, but ultimately recovered.

### Cases with second-look surgery

**Fig 3:**
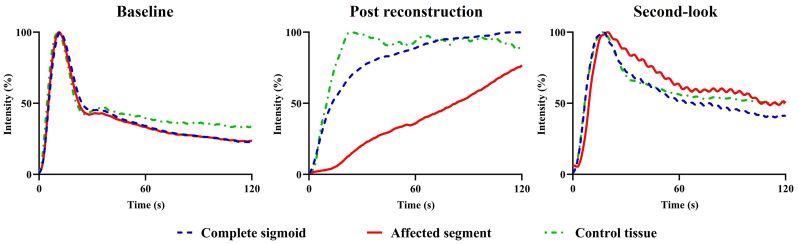
Poor perfusion pattern after fenestrated endovascular aneurysm repair (FEVAR) explantation with restoration observed during second-look surgery.

One patient underwent FEVAR explantation followed by biograft reconstruction. In this case, both qualitative and quantitative NIRF-ICG perfusion assessments indicated poor sigmoid perfusion after aortic reconstruction. To reassess the anastomoses, minimize bacterial load, and flush the surgical field, a planned second-look operation was performed 2 days after the initial surgery. During the second-look procedure, NIRF-ICG imaging demonstrated restoration of sigmoid perfusion (Fig 3). Later, the patient developed mycotic bleeding at the left iliac anastomosis, which was corrected during a third surgical intervention 12 days postoperatively. No complications related to intestinal perfusion occurred. A second patient underwent EVAR explantation and similarly underwent a planned second-look procedure. This patient demonstrated a questionable NIRF-ICG perfusion pattern after aortic reconstruction. In this patient, NIRF-ICG assessment during the second-look surgery also indicated restored sigmoid perfusion.

## Discussion

This study demonstrates that quantitative NIRF-ICG is a feasible technique for the objective intraoperative assessment of sigmoid colon perfusion during open AAA repair. Quantitative analysis revealed a consistent reduction in sigmoid perfusion after aortic reconstruction; no patient developed postoperative CI. These findings provide insight into intraoperative perfusion dynamics and highlight the capacity of colon tissue to recover from perfusion disturbances after IMA ligation.

Previous work by Tam et al^23^ reported a prolonged time to fluorescence in a patient who developed sigmoid ischemia after open AAA repair. Although promising, the study was limited by a small, homogeneous cohort, frequent IMA reimplantation, reliance on subjective visual assessment, and the absence of reference measurements. In contrast, our study applies a quantitative, parameter-based approach before and after aortic reconstruction in a more heterogeneous cohort, enabling objective assessment of relative perfusion changes over time.

The observed deterioration with subsequent recovery of sigmoid perfusion is most plausibly explained by collateral circulation through the superior mesenteric and pelvic arterial systems.^24^ The individual variability of these collateral pathways complicates reliable prediction of CI risk.^25^ Patients undergoing explantation of a prior reconstruction further illustrate this complexity. Despite a predominantly collateral-based perfusion system after the initial intervention, these patients experienced perfusion deterioration comparable with that seen after primary reconstruction. This finding highlights the multifactorial nature of perfusion dynamics in vascular watershed areas and may partly explain why IMA reimplantation has not demonstrated superiority over conventional repair.^11^ Moreover, the preservation of small-intestinal perfusion suggests that nonanatomical factors (eg, aortic cross-clamp duration, hypovolemia, and the use of inotropes or vasopressors) may disproportionately affect watershed areas, such as the sigmoid colon. Consequently, anatomical disruption alone is unlikely to fully account for ischemic events.

The two patients who underwent planned second-look procedures provide additional evidence of colon resilience. In both cases, sigmoid perfusion recovered several days postoperatively, as confirmed by NIRF-ICG imaging. Given that both patients underwent endovascular graft explantation, the transient perfusion impairment was likely attributable to factors beyond IMA ligation. Prolonged aortic cross-clamping, temporary disruption of collateral vessels, and vasopressor use are presumed to be the most relevant factors. Restoration of collateral flow, therefore, appears to be the most plausible mechanism underlying perfusion recovery.

This study is limited by including no patients who developed CI, precluding direct correlation between intraoperative perfusion patterns and ischemic outcomes. Additionally, routine postoperative computed tomography scans or sigmoidoscopy were not performed, limiting the assessment of subclinical or early-stage ischemia.^26^ The cohort predominantly comprised patients undergoing elective procedures, who are known to have a lower incidence of CI than patients undergoing acute repair, who may benefit the most from accurate risk stratification.

Despite these limitations, this study provides clinically relevant insight into the perfusion behavior of vascular watershed regions during open AAA repair. The demonstrated ability of sigmoid tissue to recover from transient perfusion deficit argues against immediate bowel resection in cases of suspected malperfusion. Instead, these findings support a strategy of heightened awareness, possibly including early use of flexible sigmoidoscopy or relaparotomy to reassess bowel viability.

Future studies should include larger cohorts with adequate representation of patients who develop CI to establish perfusion patterns predictive of postoperative ischemia. Correlation of quantitative NIRF-ICG findings with computed tomography images and sigmoidoscopy may facilitate the development of evidence-based surveillance for different stages of ischemia. In addition, systematic evaluation of perfusion variables such as hypovolemia, iliac artery stenosis, SMA and IMA patency, aortic cross-clamping, and inotrope and vasopressor use may further highlight their impact on perfusion in vascular watershed areas.

## Conclusions

Quantitative perfusion assessment using NIRF-ICG imaging during open AAA repair provides surgeons with an objective intraoperative evaluation of colon perfusion. The demonstrated ability of watershed areas to recover from transient perfusion deficits suggests that immediate resection may not be preferred in cases of suspected malperfusion and instead supports a strategy of heightened awareness. However, evidence-based clinical decision-making requires further characterization of NIRF-ICG perfusion patterns associated with subsequent CI to enable reliable risk stratification, optimize selection criteria for relaparotomy, and ultimately improve postoperative outcomes.

## Declaration of generative AI and AI-assisted technologies in the writing process

During the preparation of this work the author(s) used ChatGPT (February 2026 version) in order to improve language and grammar to enhance readability. After using this tool/service, the author(s) reviewed and edited the content as needed and take(s) full responsibility for the content of the publication.

## Author Contributions

Conception and design: RP, MK, SK, FT, PH, JB, AS, JH, AV, JS, JV

Analysis and interpretation: RP, MK, SK, PH, JV

Data collection: RP, MK, SK, LA, IP, JU, JS, JV

Writing the article: RP

Critical revision of the article: RP, MK, SK, LA, IP, JU, FT, PH, JB, AS, JH, AV, JS, JV

Final approval of the article: RP, MK, SK, LA, IP, JU, FT, PH, JB, AS, JH, AV, JS, JV

Statistical analysis: RP

Obtained funding: AV, JV

Overall responsibility: JV

## Funding

Cofunded by the PPP Allowance made available by Health∼Holland regarding the RULER and IMPULSE projects, Top Sector Life Sciences & Health, to stimulate public-private partnerships, and the RETINA-project receiving funding from the European Union's Horizon Europe research and innovation program under grant agreement number 101135529.

## Disclosures

None.

## Figures and Tables

**Supplementary Fig (online only):**
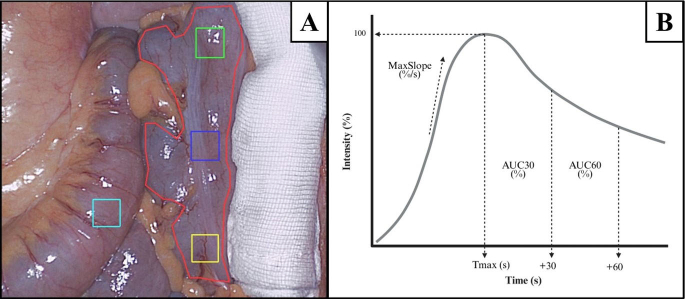
**(A)** Region of interest selection (*red*, sigmoid; *green*, proximal sigmoid; *blue*, middle segment of sigmoid; *yellow*, distal sigmoid; *cyan*, control tissue). **(B)** Schematic version of normalized time-intensity curve with derived perfusion parameters time to maximum intensity (*Tmax*), maximum inflow rate of the normalized curve (*MaxSlope*), remaining fluorescence intensity 30 seconds and 60 seconds after maximum intensity (*AUC30* and *AUC60*).
